# St. George's Hospital

**Published:** 1831

**Authors:** 


### LXVI.

### ST. GEORGE'S HOSPITAL.

IMPERFORATE ANUS, WITH MALFORMATION OF THE GENITAL ORGANS. OPERATION UNSUCCESSFULLY PERFORMED.

MANY cases of imperforate anus have been recorded: in some, operations devised and peformed for the purpose of remedying this serious deformity have proved successful, in others they have not. As these cases are not sufficiently common to be witnessed by all practitioners, the following short memoranda of one which lately occurred at this hospital may not prove altogether uninteresting.

A male child, 12 hours old, was brought to the hospital, at 6, p. m. of the 20th May, with imperforate anus. No cul-de-sac, no vestige of an outlet was to be seen, neither was any distinct impulse communicated to the finger in perinæo when the child coughed or cried. The penis, instead of being anterior to the scrotum, divided it into two equal halves, and was rather posterior to it; and the opening of the urethra was more than naturally approximated to the region of the anus. There was also a redundancy of prepuce on either side of the glans. Thus there was evidently an attempt at an anus by the change of situation in the urethra. On introducing a probe, it passed without difficulty into the bladder, and was tinged by meconium. Air, also, and urine, stained with meconium, were voided by the urethra. The child was well formed in other respects.

The case was not a promising one for an operation; but without one the child must ultimately die. The child being placed in the position for lithotomy, an incision was made by Mr. Keate in the natural situation of the anus, and a hydrocele trocar was passed in the natural direction of the rectum. It was introduced about an inch and a half or two inches, but no gut was found, no meconium flowed. The trocar was withdrawn, the canula left, and, through the canula, a probe was passed up higher than the trocar had gone. On its withdrawal, the point appeared coloured by meconium. A gum catheter was introduced into the wound with proper precautions, and retained by means of tapes. No evacuation passed through it.

The little patient passed a pretty quiet night,and sucked freely. No meconium passed through the tube. Next morning a little lukewarm water was cautiously injected, but, not returning readily, the attempt was abandoned. A stilet was passed through the gum catheter, but still no meconium issued. The child continued to take the breast, and exhibited no bad symptoms. On the following morning, a larger gum catheter was substituted for that previously in the wound, and a little more warm water was injected, but no meconium followed. This was discharged pretty freely with the urine, and the end of the gum catheter appeared a little smeared with it. In the afternoon, the infant's belly became swoln and tympanitic—it refused to suck, and in the evening it died.

On examination of the body, it was found that the rectum terminated in a conical or funnel-like manner in the inferior fundus of the bladder, just above the prostate, by a very small and nearly circular opening. The bladder itself, as is the case in young subjects, rose high above the pubes, and of course had carried the rectum up with it. The lower part of the rectum was distended with fluid meconium; its termination in the bladder was situate about an inch and a half from the perinæum. At its inferior part, nearly opposite to its termination in the bladder, the posterior wall of the rectum exhibited a small puncture, not sufficient to evacuate the contents of the gut. This had probably been made by the stilet.

The gum catheter, and in the first instance the trocar, had passed in the cellular membrane between the rectum and sacrum, pierced the upper portion of the meso-rectum, or rather the meso-colon, and lay partly in the peritoneal cavity. No viscus had been injured. There was some peritoneal inflammation, vascular injection and coagulated lymph, but not a drop of fluid was collected in the abdomen.

In this instance the trocar being passed in the *natural* direction of the gut had gone wrong, for the rectum was carried forwards and upwards towards the pubes by the bladder. At the same time, the puncture in the gut made by the stilet shews how very narrowly it had escaped the trocar and canula. In such a case at this we cannot doubt the propriety of Mr. Copland Hutchison's advice, to wait for a certain period before attempting an operation, in order that the rectum may become distended with meconium. When this is discharged by the urethra it is almost certain that the rectum is removed more or less from the sacrum, and a curved trocar following the axis of the pelvis, but directed somewhat towards the bladder, would in all probability penetrate it. In these cases of malformation, however, so many varieties occur, that a successful operation is frequently rather a lucky hit than the result of rule.

### II. RUPTURE OF THE LIVER—PERITONITIS—DEATH ON THE 13TH DAY.

Samuel Thorne, ætatis 17, was admitted into the hospital under Mr. Hawkins, at 7 a. m. of the 16th of March, 1831, having just fallen from a scaffold 30 feet in height. On admission he was in a state of collapse, cold, almost insensible. A very small quantity of warm brandy and water was given to him, and he was placed as quickly as possible between blankets. On his rallying, which soon took place, an examination was made. No injury of the ribs or thoracic viscera was detected, but the chief tenderness and injury appeared to be seated in the abdomen. At 2 p. m., he had not recovered much, but the abdomen was rather more prominent, tense, and tender. At 6, p. m. this symptom was increased, the breathing was more hurried, he lay on the left side with the knees drawn up, and the pulse had slightly risen.

*Hirud.* vj. *abdomini. Posteà fotus. H. salin, c. Tr. hyos.* ʒss. *Vin. ant. tart.* ♏xx. 4*tis. horis. Diæta febrilis.*

The pain was relieved but had returned next morning. The pulse was frequent, the tongue loaded; he had vomited in the night. *Hirud.* vj.

At 2, p. m., 12 more leeches were applied, and the patient having been sick during the day whilst the symptoms continued, six more were applied in the evening. In the morning of the 15th six leeches were again employed, and the bowels not having been opened an injection was thrown up, though with little success. In the evening six leeches; on the next morning four leeches, and an injection. The skin was now, the third day after the accident, becoming yellow, the belly was tense and full, with an appearance of deep ecchymosis on its left side, the pulse was frequent and soft. The patient occasionally vomited bilious matters. Some arrow-root was allowed, and small doses of calomel and Dover's powder exhibited every four hours. In the evening six leeches, and on the following afternoon five more. Motions were procured by injections, and on the 21st the powders were omitted. On the 22d the patient was much purged, had vomited stercoraceous matters, and exhibited aphthæ on the lips and tongue. Light bitter infusion—beef tea—arrow-root, and starch injection.

On the 25th tympanitis came on, accompanied with very distressing sense of distention of the belly. There was still occasional purging, restrainable by an opiated starch injection. On the 26th the heart was observed to be deflected a little towards the sternum, and pushed upwards, the tympanitis was distressing, the patient much reduced. He was ordered a little wine and jelly. Several attempts were also made with Weiss's and Read's syringes to pump out the air through the rectum, but without the slightest success. On the 27th he vomited nearly every thing, and said that he could swallow nothing. On the 28th he appeared to be sinking and required brandy. The sickness continued. The heart pulsated between the second and third ribs. He did not die till the 30th.

*Sectio Cadaveris.* The body was very yellow, the abdomen much distended. Some slight ecchymosis was observed in the anterior mediastinum, but no other effect of injury was detected in the thorax.

In the abdomen the intestines and other viscera were matted together by recent lymph and false membrane. In the interstices between the viscera, &c. on the right side, and in the epigastric region, was a considerable quantity of very yellow serum, with bile somewhat altered in its appearance and probably in its composition. On the left side was a considerable quantity of bloody serum, and some coagula of blood, confined also by the adhesions, &c. No rupture of any of the floating viscera could be detected.

The liver presented some ecchymosis on its concave surface, and on examination it was found to have been ruptured rather extensively. The line of laceration was confined to its right lobe and to its convex surface, and extended irregularly from behind forwards and towards the right side. One fissure, the sides of which had become perfectly united by organized blood and lymph, extended to the sulcus for the gall-bladder, but did not enter it. Another branched off to the right and blunt extremity of the viscus. Here the process of reparation was not so complete, for the injury inflicted had been greater. The sides of the laceration were wider apart, and though the uneven surface was now sealed with lymph coloured by bile, yet blood and the bilious secretion must have escaped freely by this laceration. The texture of the liver was pale throughout; there were false membranes of course around it and about it. The preparation of the liver is preserved in the museum of the hospital.

Though a celebrated author has said that a deep wound of the liver is as fatal as if it were of the heart itself, yet facts sufficiently prove that wounds of lesser severity have not unfrequently been recovered from. A bullet has been found in the gall-bladder, where it must have lodged for a length of time, and the case published by Mr. Fryer of Stamford, as well as those related by Dr. Hennen and others are sufficient evidence of the fact, as we stated it. There is nothing very astonishing then in the present patient's having survived for 14 days, but the case is interesting as displaying the symptoms occasioned by this dangerous accident, and the dissection is interesting also as exhibiting a good instance of the reparative efforts of nature.

### III. INJURY OF THE LUNGS—RECOVERY.

FRANCES WHITE, æt. 13, was admitted into the hospital under Mr. Hawkins, at 6, p. m. of the 6th April. Half an hour previously she had been run over by a cabriolet, but neither she nor those who brought her could give any account of the precise manner in which she was injured. On her admission she was rather low, but not in a state of absolute collapse. She complained of pain in the chest, but did not refer it to one side in particular. There was some tenderness on pressure at the junction of the false with the true ribs on the left side, but no fracture could be distinguished. She had lost a little blood from the nose.

At 10, p. m. the pulse was weaker, the extremities were cold, there was collapse, with great pain in the chest.

*Mist. æth.* ℥ss. *Liq. opii sedat.* ♏x. *M. o.* ½ *horâ dum perst. collapsus.*

7th, 2, a. m. Reaction. Complains of great pain in left side of chest, extending to the spine, with cough, and hurried breathing. Says she had cough before the accident.

*V. S. ad* ℥x. *Omr. haust. Æth. comp.*

4, a. m. Was relieved by the bleeding, but pain is still considerable—pulse weak.

*Haust. Liq. ammon. acet. c. Vin. ant. tart.* ʒss. *Tr. hyosciami,* ʒss. 4*tis horis. Potus frigidus tantummodo.*

10, a. m. Dyspnœa and pain increased—cough continues—generally lies on her left side with the knees drawn up—spits globular masses of blood rather florid, and mixed with air-bubbles. Pulse has again risen—bowels not yet opened.

*V. S. ad* ℥viij. *Haustus sennæ,* ℥iss.

6, p. m. Was relieved by the bleeding, and the pulse was kept down by it until this afternoon, when it again began to rise. Much pain and dyspnœa——tongue moist—bowels only scantily opened.

*V. S. ad* ℥vj.

10, p. m. The blood taken this morning and at 6, p. m. was cupped and shewed some buff. That drawn at first of course did not. Has again exacerbation of pain, and the pulse has risen—bowels not open.

*V. S. ad* ℥vj. *Hyd. sub. Pulv. ant. Pulv. scammon. sing.* gr. v. *statim. Haust. sennæ post horas tres.*

8th, 8, a. m. Was relieved by the bleeding. One cup shews but slight and nearly transparent buff, and loose coagulum. Still spits small globular masses of blood, now mixed with a little bronchial mucus—much pain in left side—pulse frequent, without force, tongue rather white—bowels not opened.

*Hirud.* x. *lat. sinistro. Haust. sennæ.*

10, a. m. Vomited her draught—bowels not opened. Was relieved by leeches, but now looks depressed.

*Enema avenæ tenuis c. H. sennæ,* ℥iss.

10, p. m. Was much better this afternoon, and spat merely mucus very little blood-stained. This evening a fresh attack of pain came on, and there is now a febrile exacerbation.

*Hirud.* viij. *lat. sinistro.*

9th, 4, a. m. Still complains of great pain.

*Adde haustui salin. anteà prescripto tr. camph. c.* ʒss.

9th, 4, a. m. Still much pain—more mucous blood-stained expectoration—pulse more frequent—skin warm—tongue moist—bowels not open.

*Hirud.* viij. *lat. sinistro. Enema Aven. c.* ℈j. *H. sennæ,* ℥ss. *Calomel,* gr. iv. *Pulv. jalap.* gr. xij. ♏. *h. n. s.*

10th. Bowels were freely opened by powder—less frequency of pulse and heat of skin—has suffered and still suffers from much pain in the back and *right* side of the chest.

*Adde haustui Syrup. papav.* ʒj.

*Vesp.* Pain still continues.

*Fotus assiduè c. Dec. papaverum.*

11th. Pain much relieved—still cough—expectoration muco-purulent, not very copious and rather bloody.

*Broth—arrow-root.*

On the next day she was allowed some beaf-tea, and on the 13th she appeared convalescent, the sputa being merely mucous and free from blood. On the 15th she had a fresh accession of pain in the chest, dyspnœa, cough, and expectoration of brown and evidently blood-coloured mucus. The blood did not appear to be recent, but rather to have been effused for some little time in the substance of the lung; it had no offensive smell.

*V. S. ad* ℥vj. *(she fainted on* ℥iij. *being taken.) P. c. Haust. ut antea,* 6*tis horis.*

2, p. m. *Hirud.* x. *lat. dextro.*

*Vesp.* Pain and other symptoms relieved by the depletion—rather sick from the medicine.

On the 17th the expectoration was trifling, and not decidedly bloody; the symptoms of inflammation were removed. On the 17th she was allowed some beef-tea, on the 19th a chop and the draught was only given twice daily. She gradually recovered, was ordered some sulphate of quina on the 29th, and on the 10th of May was discharged the hospital. At this time she had scarcely any cough, but looked pale and very delicate, and did not expand her chest very perfectly. We regret that we made no stethoscopic examination. Several of the girl's family had died of phthisis.

We have given a full report of this case, because it seems to shew, in a clear manner, the progress of symptoms, and the influence of treatment. That the lung on the left side was more or less ruptured we suppose there can be no question, nor can any doubt exist of the supervention of pulmonic inflammation. The case is interesting in a practical point of view, and the result is satisfactory. If the injury was not so extensive, nor the inflammation so intense, as in many of the instances recorded in the annals of military surgery, yet the patient was a delicate child of a strumous habit and with a phthisical tendency, to whom the abstraction of half a pound of blood would prove as serious a loss as that of half a dozen to a robust young soldier, or a hardy veteran. It is a curious, but by no means an inexplicable fact, that wounds of the thorax have always been found comparatively seldom followed by fatal consequences. Dr. Gregory was in the habit of stating in his lectures, that of twenty-six wounds of the thorax received at the battle near Quebec, two only were fatal. There are two lungs, and when one is injured, the other may be made to ease it of much of its duty; besides which, the lung is so vascular, supplied so peculiarly and directly from the venous system, that in depletion from that system promptly begun and boldly pursued, we possess an agent of the utmost power in controlling the traumatic inflammations of the organ.

### IV. SIMPLE FRACTURE OF THE LEG—TRAUMATIC DELIRIUM — DEATH — ULCERATION OF THE BOWELS.

EDWARD EAMES, æt. 52, was admitted under Mr. Keate, at midnight, of the 30th December, 1830. It was during the severe frosty weather, and the accident had happened from his falling in the street. There was oblique fracture of the left tibia a little above the ankle-joint, and the fibula was broken somewhat higher. The integuments were not lacerated, but they were put upon the stretch. The patient was collector to a brewery, had apparently lived freely, and was of a remarkably irritable temperament.

*Leg placed in a junk—cold lotion—Liq. opii sedativ.* ʒj. *stat.*

31st. *Vesp.* Got very little sleep last night. Is fidgetty and morbidly alive to what is going on around him—skin cool—pulse rather irregular, slow, full —tongue moist; has been sick twice.

*Beef-tea. Gin,* ℥j. *in water. Liq. op. sed.* ♏xxv. *Sp. junip. c.* ʒiss. *Mist. camph.* ʒxj. *M. h. s. s.*

In the evening of the 1st January he was in much the same state, talked much about his family, and had been twice sick. The gin and draught were repeated. At one o'clock next morning he was suddenly seized with a paroxysm of such violent delirium, that it required several men to hold him down. He had not slept an instant since the preceding night; before he could be secured he had nearly torn off the junk apparatus.

*Liq. op. sedat.* ʒj. *stat. Strait-waistcoat, &c.*

He continued in a very restless state, talking incessantly till 4, a. m. after which he had some sleep at short intervals. During sleep the breathing was stertorous, as if he were under the influence of opium. The delirium, it should be stated, partly hinged on things present, and partly on things past.

10, a. m. *Liq. op. sed.* ♏x. *Ammon, carb.* gr. vj. *Mist. camph.* ʒxij. *M. 4tis horis.. Gin,* ℥iss. *in water.*

During the day he was quieter, and at night an anodyne and the gin were repeated. During the third, he continued free from delirium, but his manner was still quick and his eye restless. The bowels not having been opened since the occurrence of the accident, he was ordered some aperient pills, with 4 ozs. of gin, and the night draught. He passed an unquiet night. Next morning his bowels were freely opened by a senna draught. At 11, a.m. he was in a state of considerable excitement, and had torn off the junk apparatus.

*Liq. op sed.* ʒj. *stat., Aug. dos. Liq. op. sed. in haust. quotidian, ad* ʒss. *Liq. op. sed.* ʒj. *Sp. æth. sulph. c.* ʒj. *c. Mist. camph. h. n.*

The delirium continued during the evening, but during the greater part of the night he was under the influence of the opium. He slept much, only awaking at times, when he was very restless, though not so delirious as before. On the fifth he was quieter, though still inclined to delirium; the pulse was soft and slow, the tongue moist.

*P. c. haust.* 6*tis horis, et adde Sp. ammon. arom.* ʒss. *Sing. haust. H. anod. h. n.*

In the evening he was much more composed and rational. He had been sick two or three times during the day, the matters vomited being dark and acid—he perspired a good deal—tongue moist—pulse slow. He passed a pretty tranquil night, and on the 6th there was little hurriedness of manner. He had been very sick once or twice during the night.

*Moschi optimi*, gr. v. *Ext. opii*, gr. j. *M.* 6*tis horis. Om. alia præter genevam.*

He continued to be sick during the 6th, and in the evening the pulse was rather more frequent—tongue white in the centre—disposition to perspiration—bowels not opened since the 4th. An effervescing draught, with rhubarb and calcined magnesia, was ordered to be taken occasionally, and for a time it checked the sickness. On the 7th his manner was almost natural, and after this day he suffered no more from delirium. A mustard poultice was applied to the epigastrium, after every fit of vomiting. This however continued, and on the 8th and 9th was conjoined with looseness of the bowels approaching in character to dysentery, but without blood in the motions. There was some obscure tenderness in the belly. The quantity of gin was diminished to ℥iv. and the same quantity of red wine was exhibited.

12th. For the last three days there has been little alteration of the symptoms. The vomiting has continued more or less, appearing at times to be checked by a little burnt brandy, but continuing at others in spite of it. The mustard poultices have had little effect. He has occasionally purging, and frequently attempts to void a stool without effect; the motions are very watery and offensive. The tongue is disposed to be dry and red at the sides and tip; the pulse is more frequent, and there is still a slight febrile exacerbation in the evening. There is still the same obscure tenderness about the belly without tension. No delirium. It was supposed, from the character and continuance of these symptoms, that there was some inflammation of the mucous membrane of the bowels, and probably ulceration. The stimuli were now finally abandoned, and small doses of hyd. c. cret. with James's powder and hyosciamus were prescribed, a blister applied to the umbilicus, with subsequently some leeches to the hypogastrium, and small starch injections to check the tendency to diarrhœa. Arrow-root, sago and the most unirritating farinaceous food were ordered. We need scarcely pursue the details of the case, suffice it to say, that though the strength declined the symptoms never varied in character. The vomiting continued, with a disposition to purging,—there was a well-marked febrile exacerbation in the evening, but up to the very moment of his death there was not even a tendency to delirium. At 11, a. m. of the 17th he died.

*Sectio Cadaveris;* 28 *hor. post mortem.* Body still corpulent, but wasted, when compared with the patient's state on admission.

*Cranium.* Dura mater very adherent to the calvarium—no perceptible morbid alteration of the membranes or structure of the brain. The latter firm, and containing very little fluid in its ventricles. Coats of the carotids exhibiting the opacities so common in advanced life.

*Thorax.* Lungs very healthy—heart large, flat, flabby. No material alteration of the great vessels.

*Abdomen.* No peritoneal inflammation. Liver inclining to the nutmeg character, without distinct granular deposit. Kidneys hard, not otherwise unhealthy — bladder so contracted as scarcely to hold half an oz. of urine. Stomach marbled at its cardiac end with large congested veins—no unusual vascularity of its mucous membrane, which was rather pulpy. Lower portion of the jejunum, and upper of the ileum of a deep and blood red dye in patches of from several inches to a foot in length; the vessels and capillaries being gorged and injected with blood. At intervals ulcers of small size not clustered together, chiefly disposed on the valvulæ conniventes, some with a raised and vascular margin, some without it, none displaying an attempt at cicatrization. In short, the mucous membrane in a state of inflammation, which has terminated in scattered ulcerations. Peritoneal coat perfectly healthy.

The leg was in surprisingly good position, when the restless state of the patient is considered. The process of union had made some progress.

The fate of this patient is certainly rather curious; he died of a "veritable gastro-enterite." The delirium entirely subsided under the treatment employed, and it would be difficult to pronounce upon the causes of the inflammation of the mucous membrane of the bowels. The quantity of stimuli was never excessive, scarcely a tithe of what has been exhibited with success in cases of this description. The utmost quantity of liq. op. sed. which was taken in 24 hours amounted to ʒiv. One circumstance is perhaps deserving of consideration. The patient having lived freely had of late suffered much from that malady which free living is so admirably calculated to produce—severe dyspepsia. For this he had liberally employed both physic and gin, and this condition of the intestinal tube might possibly have contributed to light up that inflammation and ulceration which occasioned his death.

### V. INJURY OF THE SPINE—TEMPORARY PARALYSIS OF THE BLADDER, AND NUMBNESS IN ONE LIMB.

Rosa Albedine, æt. 19, was admitted at 2, a. m. of the 27th April last, under the care of Mr. Keate. She had jumped from a second floor window, 30ft. from the ground, and she thought that she had fallen directly on her back.

There was not much general collapse. She could not move the right lower extremity to any extent, and complained of numbness in the right thigh and hip. She complained also of pain in the situation of the spinous processes of the lower dorsal and upper lumbar vertebræ, and though no fracture could be felt there, a slight projection of the last dorsal spines seemed present.

At 11, a. m. the numbness of the right thigh was rather increased, and she could bear to be forcibly pinched without experiencing inconvenience. There was much pain in the back, with incapability of turning in bed. The skin was warm and moist; pulse frequent, not hard; bowels not open. She could not pass her urine, and when drawn off by the catheter it was very dark, abundant, acid.

*C. c. lumbis ad* ℥x. *H. sennæ. D. feb.*

The urine was drawn off again in the evening and had the same characters. On the 28th the numbness was rather increased and extended below the knee. She complained much of pain in the right iliac and costal regions, the bowels were not open, the urine required to be regularly drawn off.

*Hirud.* xiv., *lat. dolenti. H. sennæ,* ℥iss., *et rep. o. 2ndâ hor. donec alv. respond.*

On the 29th the pain was relieved, there was little pyrexia, the numbness was diminished, and she had once made water without the assistance of the catheter. Some saline draughts were given, and low diet continued. After this she progressively improved. The numbness of the thigh passed away, the power of motion of the limb returned, and the urine could be voluntarily passed. As the numbness of the thigh disappeared the patient began to suffer a good deal of pain in it, and a blister was ordered on the 6th May. To the application of this she refused to submit, and left the hospital, since which we have heard nothing more about her.

### VI. ABSCESS, APPARENTLY IN THE LIVER—PUNCTURE WITH A TROCAR—ULCERATION AND SLOUGHING AROUND

THE PUNCTURE — DEATH — DISSECTION.

William Hollock, æt. 31, formerly a seaman, was admitted Dec. 23, 1830, under Dr. Seymour, from whom he was shortly afterwards transferred to Mr. Hawkins.

In the right hypochondrium, beneath the cartilages of the 6th, 7th, and 8th, ribs, a tumour of small size, fluctuating obscurely, apparently depending on abscess in the substance of the liver, which is felt in the epigastrium. Pain on pressure of the tumour or integuments in the neighbourhood — some erythematous redness of the skin — complains of pain in the tumour and in the right shoulder. Countenance sallow, anxious—cough—some emaciation — disposition to perspiration at nights.

Three years ago, whilst in the Navy, he had severe liver-complaint in Bengal, for which he was salivated. He recovered, came home, and turned plasterer, which he has since been. Present complaint commenced seven weeks ago with acute pain in the right hypochondrium, cough, and fever. He has been bled seven or eight times, blistered, and salivated. Has observed the tumefaction for about a month; has had no shivering. A poultice and an anodyne composed the treatment, till the 15th of January, when the tumour had become more prominent, and fluctuation, though still obscure, was more distinct. A trocar was introduced some two or three inches, and from two to three ozs. of thick dirty-looking pus were slowly drawn off through the canula. A portion of gum catheter was secured in the wound, and for a few days the matter continued to drain out through it. The catheter then slipped out, and was never replaced. The patient suffered little from the operation, but felt relief from the discharge of pus. A little porter and red wine were allowed, the matter continued to drain through the puncture, and for a little while all went well. On the 28th the matter appearing to be rather confined the opening of the abscess was enlarged with a lancet. After that there was a disposition to bleed in the cyst, and on pressing the parts around, on coughing, or on deep inspiration, coagula were squeezed out of the opening.

For some days he complained of severe pain in the left elbow and forearm, and of incapability of moving the thumb and fore-finger. After a time, however, these symptoms subsided under the employment of frictions with stimulating liniments. On the 18th Feb. the margins of the opening appeared disposed to ulcerate and looked sloughy, with fungous granulations at its mouth. There was some fulness in epigastrio near the wound, with redness and thinning of the integuments. He had little cough and no purulent expectoration; he could not sleep at nights. He was ordered laudanum, but it disagreed with him, and the 4th of a grain of acetate of morphia was substituted for it with good effect. On the 22d the puffy fulness in the epigastrium having increased, a puncture was made, and a little curdly pus and more blood discharged. After this he improved for a time, and gained strength and flesh. The discharge from the openings, though still bloody, diminished, the tumefaction around decreased, and the openings themselves shewed a disposition to contract. On the 13th March the wounds were nearly closed, but a thin bloody discharge still issued from the original one. He complained much of flatulence, some hiccup, and pain in the situation of the diaphragm on taking a deep inspiration. On the 15th one-third of a grain of morphia was given every night. The wound now began to ulcerate, and a sore with a foul sloughy surface resulted. The sore partly sloughed and partly ulcerated, and dirty-looking slimy coagula were in its centre. The integuments around were blueish, with induration and tenderness. There was not much constitutional disturbance, but great irritability. In fact the patient had always been remarkably fretful and irritable.

*Cataplasma dauci. Haust. liq. ammon. acet. c. Morph. acet.* gr. ⅓d. *Ammon. carb.* gr. vi., 6*tis. horis.*

For a day or two the sore improved in appearance, but then it degenerated again into the foul sloughy ulceration. Several applications were tried without effect, and gin and wine were freely given. The quantity of morphia was gradually increased until it amounted to one grain every six hours. By this dose he was fairly brought under the influence of the preparation, yet still the sore gained in size, and on the 12th of May was 4 or 5 inches in length, in a direction *across* the belly, and 3 or 4 in breadth from above downwards. The sore was superficial, affecting chiefly the cutaneous and subcutaneous tissues; its characters we have described before; in its shape it shewed a disposition to circularity, like the true hospital ulcer. The undiluted nitric acid was tried, but it gave great pain, and on the separation of the slough the sore had spread. The weak nitric acid lotion, laudanum, chloride of soda, in short a variety of applications were tried, but though some occasioned less pain than others none checked the progress of the sore. It spread with a widely diffused blue induration of the circumjacent skin; at its border was a thin margin of granulation, situate in the cutis itself, for the subcutaneous cellular membrane and the fascia sloughed without an attempt at reparation. The surface of the sore was one slimy dirty yellow slough, its circumference a broad halo of dark inflammation. The pain and tenderness were chiefly in the inflamed integuments around. The constitutional symptoms were those of low irritation; a pulse scarcely to be felt; a skin bedewed with a clammy moisture; a white tongue; and a sunken countenance. Before the patient's death this peculiar and frightful looking sore had spread over the belly beyond the umbilicus, and on the other side beyond a perpendicular line drawn from the anterior spine of the ilium upwards to the ribs. He died exhausted, not by the discharge, for that was not profuse, but by the irritation. Before death he vomited good bile, and his stools were of good colour. He had no symptoms of disturbance of the lungs, few of disturbance of the cerebrum. He expired on the 12th of June.

*Sectio Cadaveris*. June 14th. Body a good deal emaciated.

*Cranium*. Not examined.

*Thorax*. Lungs generally sound. Some adhesions of lung to diaphragm on right side, with a very slight quantity of serum in the chest. Lungs healthy. Heart and great vessels healthy.

*Abdomen*. Liver united by old adhesions to the abdominal parietes, for some space opposite the sore. Through the opening formerly made by the trocar, now closed by soft slough, a probe passed down to the cavity of an abscess somewhat larger in circumference than a walnut, but not so deep. This abscess did not appear to be in the liver, but rather on its surface. There was indeed opposite the puncture a yellow discoloration, as of cicatrix and obliteration of natural structure by lymph, penetrating the liver for about an inch. This probably had been the spot where the trocar had entered the substance of the liver. Liver around perfectly healthy. Abdominal viscera sound.

The abscess had probably not been in the substance of the liver, but on its surface. The liver might have been inflamed in India, but of an Indian hepatitis it bore no mark. From what appeared on the dissection it is more reasonable to conclude that the peritoneal surface of the liver had been inflamed, that suppuration had followed, that the matter had been confined by adhesions around it, and that a circumscribed abscess had resulted, than that one had been formed in the substance of the liver, which was otherwise healthy, and left no trace behind it. To this explanation Mr. Hawkins yielded his assent. We presume that none will doubt the propriety of Mr. Hawkins' practice. He did all that a surgeon could do under existing circumstances; and with all the knowledge acquired by dissection we do not know that he could have done more, and we scarcely see how he could have done less. Perhaps it might have been better if no foreign body had been left in the wound, but on this point different persons may form different opinions.

The nature of the sore which followed the puncture was peculiar; its causes are involved in difficulty. In its characters and progress it certainly approached to those of the hospital sore, the "pourriture d'hôpital," which was often witnessed at the Hôtel Dieu, and more lately seen in the military wards at Bilboa. It was not so rapid in its progress, neither were there any other sores of a similar description in the house, and therefore we must refer it to some peculiarity in the habit or in the part, in other words, we must confess that we know nothing about it.

---

